# Concurrent associations between mothers’ references to internal states and children’s social understanding in middle childhood

**DOI:** 10.1111/sode.12356

**Published:** 2019-01-24

**Authors:** Amy L. Paine, Salim Hashmi, Siwan Roberts, Rhiannon Fyfield, Dale F. Hay

**Affiliations:** ^1^ School of Psychology Cardiff University Cardiff United Kingdom; ^2^ Child and Adolescent Mental Health Service (CAMHS) Bangor University Bangor United Kingdom

**Keywords:** community sample, family relationships, internal state language, longitudinal study, social understanding

## Abstract

Although it is well established that features of maternal speech are associated with children’s social understanding in the preschool years, few studies explore this relationship in middle childhood. Within the context of a prospective longitudinal study of a representative community sample of families (subsample *n = *207, mean age = 82.88 months), we investigated concurrent associations between mothers’ internal state language and aspects of 7‐year‐olds’ social understanding, including children’s understanding of belief and spontaneous references to internal states during free play. When sociodemographic, maternal, and child characteristics were controlled, mothers’ references to their own cognitions were associated with dimensions of children’s social understanding. Our findings suggest that exposure to others’ perspectives contributes to children’s advanced understanding of minds, which has implications for interventions that foster social understanding.

## 1 INTRODUCTION

Many features of children’s early conversational environments have been explored in relation to the development of social understanding. Children’s early conversations, particularly mothers’ references to internal states, are associated with children’s understanding of minds (Tompkins, Benigno, Kiger Lee, & Wright, 2018). However, the mother–child relationship is renegotiated continuously as the child undergoes physical, social, behavioural, and cognitive changes (see Collins & Russell, 1991), and so the strength of associations between mother–child interaction and children’s outcomes is not consistent over time (Bradley, Pennar, & Iida, 2015).

There is limited research on later developments in children’s understanding of minds, despite middle childhood being a developmentally appropriate time to study advancements in social understanding. In this age period, children increasingly spend more time outside of the home as they navigate relationships at school and acquire a more sophisticated understanding of other people’s minds (Hughes, 2016). However, only a few studies explore the ongoing importance of mother–child interactions for older children’s social understanding (e.g., Ensor, Devine, Marks, & Hughes, 2014). We addressed this gap in the literature by investigating contemporaneous associations between mothers’ speech and measures of children’s understanding of minds in middle childhood.

### 1.1 Maternal references to internal states and children's social understanding

*Internal state language* (ISL) encompasses references to an individual’s internal, mental world (e.g., references to desires, intentions, emotions, and cognitions). Although mothers’ general propensity to use ISL tends to remain stable over time (Ruffman, Slade, & Crowe, 2002), as children develop, mothers use more ISL and increasingly refer to more abstract internal states, such as cognitions (e.g., thoughts and knowledge; Beeghly, Bretherton, & Mervis, 1986). Mothers’ references to cognitions are the most consistent predictor of preschool‐age children’s social understanding (Tompkins et al., 2018). This aspect of mothers’ speech is related to children’s developing abilities to think about and discuss thoughts and knowledge (Beeghly et al., 1986) and may give children particular awareness and insight into how the mind works (Booth, Hall, Robison, & Kim, 1997).

Ongoing investigation of factors that influence children’s more advanced understanding of minds in middle childhood is crucial, given that older children’s social understanding is associated with numerous positive outcomes (Caputi, Lecce, Pagnin, & Banerjee, 2012). However, although positive associations between mothers’ references to cognitions and children’s social understanding skills are well established for preschool‐age children, few studies explore the ongoing importance of this dimension of mothers’ speech beyond the fifth year of life (Ensor et al., 2014). In one study, mothers’ references to cognitions during interactions with their 6‐ and 10‐year‐old children were associated with children’s understanding of minds (Ensor et al., 2014). Further research is warranted to advance our understanding of the conditions and processes by which mothers’ current references to cognitions might influence children’s more advanced social understanding in middle childhood.

Analysis of the *referent* of mothers’ references to cognitions—whether they are referring to their own cognitions or those of their children—may reveal processes whereby mothers’ ISL fosters social understanding skills (Adrian, Clemente, & Villanueva, 2007). In early childhood, mothers’ talk about the *child's* internal states predicts children’s later references to desires and emotions (Taumoepeau & Ruffman, 2006), which suggests that children’s social understanding skills are fostered by caregivers’ speech that encourages children to attend to, reflect on, and represent their own states of mind. According to prior research, mothers may introduce mental states to children in the preschool years primarily by commenting on children’s own internal experiences, supporting the possibility that children first learn to understand or predict the behaviour of others based on their understanding of their own inner states, enabling children to imagine (or “simulate”) the thoughts of others (Taumoepeau & Ruffman, 2006). However, at later ages, mothers’ ISL may foster children’s understanding of minds via different processes (Adrian et al., 2007). As children develop, mothers increasingly discuss other people’s internal states (Beeghly et al., 1986), providing children with opportunities to appreciate the perspectives of others (Harris, 1999). As such, by middle childhood, mothers’ references to *her own* inner states may foster children’s ability to understand the minds of others. Taken together with the established importance of mothers’ references to cognitions in middle childhood (Ensor et al., 2014), we expected that mothers’ references to *her own* cognitive states might be particularly important for the development of children’s ability to think about the thoughts of others in middle childhood.

Thus far, many studies of children’s social understanding in middle childhood have focused on children’s performance on *strange stories *tasks, which measure children’s advanced insights about the mind, such as understanding of deception and misunderstanding (Ensor et al., 2014). We aimed to extend knowledge about social understanding in this age range by employing additional age‐appropriate tasks to measure children’s ability to think about the thoughts of others. Children’s understanding of *second‐order false belief *assesses children’s understanding that one person can have a mistaken belief about *another person's* belief. In contrast to first‐order false belief tasks that assess children’s understanding of what people think about reality, successful interaction with others often depends on being able to think about other people’s thoughts (Perner & Wimmer, 1985). The second‐order false belief task is a developmentally appropriate assessment of advanced understanding of minds (Perner & Wimmer, 1985) and part of the developmental sequence of children’s understanding of false beliefs (Hayashi, 2007).

Children’s spontaneous references to the thoughts and knowledge of others may also reveal more details of what children understand about other people’s minds. Though children’s propensity to use ISL is positively associated with other measures of social understanding in the preschool years (Ruffman et al., 2002), the evidence for this association is mixed in middle childhood (e.g., Longobardi, Spataro, & Renna, 2014). This may be because children’s ISL is often assessed within written tasks rather than free play, which is a rich context for children’s spontaneous speech about internal states in middle childhood (Howe, Abuhatoum, & Chang‐Kredl, 2014).

Furthermore, when analysing children’s speech, their references to thoughts and knowledge may be a better marker of their understanding of other people’s thinking than their general tendencies to make references to internal states (Howe et al., 2014). Over the preschool years and beyond, children increasingly refer to others as beings with thoughts and knowledge (Booth et al., 1997). Given that references to others’ internal states are associated with perspective‐taking skills (Howe, 1991), it is also important to take into account the referents of children’s sponataneous speech about cognitions. Therefore, children’s speech about cognitions was analysed by referent.

### 1.2 Family, maternal, and child characteristics and social understanding

Any association between mothers’ current references to cognitions and children’s social understanding might be explained by other factors, such as sociodemographic risk and family conversational styles. Sociodemographic disadvantage is associated with poorer child performance on social understanding tasks (Cole & Mitchell, 2000) and with less maternal ISL (Howard, Mayeux, & Naigles, 2008). Mothers’ general talkativeness is positively associated with mothers’ production of ISL (Ensor & Hughes, 2008).

Additionally, when seeking evidence for the importance of mothers’ current references to internal states for children’s social understanding, mothers’ past tendencies to talk about internal states should be taken into account. Mothers’ propensity to refer to internal states shows stability over 1 and 2 years (Ruffman et al., 2002), and as such is characterized as a cognitive‐behavioural trait (Meins, Fernyhough, Arnott, Turner, & Leekam, 2011). Possibly, mothers’ references to internal states in middle childhood simply reflects their earlier tendencies to talk about people’s inner worlds rather than exerting direct influence on the development of more advanced social understanding.

Finally, any association between mothers’ ISL and children’s social understanding in middle childhood could be explained by other characteristics of the child. The child’s age and language ability are both associated with children’s understanding of second‐order false belief (Paine, Pearce, van Goozen, de Sonneville, & Hay, 2018). Given that mothers tend to be more talkative with their daughters than with their sons (Leaper, Anderson, & Sanders, 1998), the child’s gender might also be associated with mothers’ references to internal states. There is also mounting evidence for the ongoing influence of working memory on social understanding in middle childhood (Lecce, Bianco, Devine, & Hughes, 2017). Finally, it is certainly possible that cognitively able and verbally precocious children might elicit more ISL from their mothers; therefore children’s own references to internal states *within* the mother–child conversation must be taken into account.

### 1.3 Aims of the study

We investigated mothers’ references to internal states and features of children’s social understanding in middle childhood within a moderately sized, representative sample of British families. We aimed to test the hypothesis that mothers’ references to internal states while interacting with their children were associated with measures of advanced social understanding skills. Specifically, we hypothesized that mothers’ references to her own cognitions would be associated with (a) children’s understanding of second‐order false belief and (b) children’s references to others’ cognitions during free play. By controlling for sociodemographic, maternal, and child characteristics, we tested alternative hypotheses that any concurrent associations between maternal speech and these measures of social understanding in middle childhood could be explained by sociodemographic characteristics, the mothers’ verbal behaviour (including their earlier references to internal states), or various characteristics of the child.

## 2 METHOD

### 2.1 Design

The Cardiff Child Development Study (CCDS) is a prospective longitudinal cohort design that investigates children’s early social development in a nationally representative British sample of mothers and their firstborn children. Three hundred and thirty‐two primiparous women and their partners were recruited from National Health Service (NHS) antenatal clinics in hospitals and GP surgeries in Cardiff and The Vale University Health Board, and the Gwent Healthcare Trust, U.K. Data collection took place in pregnancy and at a mean of 6, 12, 21, 33, and 84 months postpartum. Ethical approval was obtained for the procedures from the NHS Multi‐Centre Research Ethics Committee and the Cardiff University School of Psychology Research Ethics Committee.

### 2.2 Participants

The present study focuses on the second and sixth waves of the study, which were both home visits scheduled when the firstborn child was between 5 and 7 months of age (the *early infancy *assessment; *M = *6.64, *SD *= 0.88) and between 6.5 and 7.5 years of age (the *middle childhood* assessment; *M = *83.28 months, *SD *= 4.54), respectively. In early infancy, 310 families (94%) were assessed, with 301 observed at home and 9 providing questionnaire data only. Six families had withdrawn from the study; eight were not traced; four cancelled appointments and could not be rescheduled; and four remained in the sample but could not be assessed due to health problems or family complications.

By middle childhood, 22 of the 332 families recruited in pregnancy had withdrawn from the study and one family had never been traced, leaving 309 (93%) remaining in the study. Of those, 287 (93%) provided data at the middle childhood assessment: 15 were only able to complete questionnaires; 14 had partial home visits; two had equipment failures; one family withdrew their data after testing; and one child could not be tested due to a severe developmental delay. Of the 287 children who were assessed, 254 were video‐recorded interacting with their primary caregiver (228 with the mother, 22 with the father/stepfather, 4 with a grandmother). The present study focuses on 207 of the mother–child dyads (*M *age = 82.87 months, *SD *= 4.44) filmed in the interaction task at the middle childhood assessment who had also been filmed in the interaction task at the early infancy assessment.

### 2.3 Procedure

#### 2.3.1 Early infancy assessment

The early infancy home visit took approximately 2 hr. During that time, the mothers were interviewed and a questionnaire battery was administered to mothers, fathers and, where possible, another significant person in the infant’s life (such as a close family member or friend). The infant was filmed during a 25‐min assessment where various social, emotional, and cognitive tasks were administered, including several parent–child interaction tasks. Upon completion of the assessments, a remuneration of a £20 gift voucher was given to the family.

#### 2.3.2 Middle childhood assessment

Families were visited in the home by two or three research assistants for two 2‐hr sessions (*M* age at session 1 83.21 months, at session 2 83.88 months). While the primary caregiver completed an interview with one trained research assistant, the child completed various cognitive, social, and emotional assessments in a quiet space with a second trained research assistant. At the end of each session, the child and caregiver took part in a mother–child interaction task and some family games. When required, a third research assistant accompanied the research team to keep younger siblings from interfering with the child testing and mother–child interaction tasks. A remuneration of £20 was given to the caregiver and a book voucher of £10 to the child at the end of the home visits.

### 2.4 Measures

#### 2.4.1 Sociodemographic adversity

Information about fathers was not present in all cases; therefore the sociodemographic adversity index was based on maternal characteristics collected during pregnancy and early infancy. The child’s exposure to *sociodemographic adversity* was indexed by the following risk variables: (a) *Social class*, a dichotomized variable indicating individuals as working class or middle class, based on the highest rank of employment the mother ever had at entry into the study on the Standard Occupational Classification 2000 (SOC2000; Elias, McKnight, & Kinshott, 1999; 55.9% were middle class); (b) *Maternal education*, a dichotomized variable indicating whether mothers had achieved the minimum level of qualifications required for the completion of secondary education in the U.K. (General Certificate of Secondary Education examinations grade A*‐C or equivalent; 81.9% achieved); (c) *Young maternal age*, dichotomized according to whether or not mothers were in their teens at the time of birth of the child (mean = 28.6 years); (d) *Stable partnership with the baby's father *(i.e., married, cohabiting, or in a committed relationship but not currently living together; 91.3% were in a stable partnership) and (e) *Parents legally married* were also dichotomized (53.5% were married). All these items were categorical; therefore, a principal components analysis based on the polychoric correlation matrix confirmed that all these items contributed to a single component, which explained approximately 77% of the shared variance in these risk indicators. The single summary score derived from this PCA measured the family’s exposure to sociodemographic adversity (Perra, Phillips, Fyfield, Waters, & Hay, 2015); positive scores indicated a higher than average exposure to risk factors for sociodemographic adversity. The mean score for sociodemographic adversity in the present subsample was −0.15, (*SD *= 0.92, range = −0.95 to 2.51). The 207 families in the present sample were not significantly different from the original *N* = 332 recruited with respect to sociodemographic adversity scores, *t*(465.57) = −1.82, *p* = .07.

#### 2.4.2 Mother's verbal ability

In the second visit of the middle childhood assessment, the *Wechsler Test of Adult Reading* (WTAR; Wechsler, 2001) was used to assess *mothers’ verbal ability.* Data were available for 184 (89%) of mothers with interaction data. Each mother’s score was calculated by age‐normalizing the data to produce a standardized score. The mean score for mothers’ verbal ability was 97.92 (*SD = *14.20) and ranged from 55 to 122 out of a possible score of 129.

#### 2.4.3 Child's verbal ability

In Visit 2, children’s receptive vocabulary was assessed using the *British Picture Vocabulary Scale* (BPVS; Dunn, Dunn, Whetton, & Pintilie, 1982) as an estimate of *verbal ability*. Within the sample of children who had mother–child interaction data available, 205 (99%) completed the assessment. The mean standardized score for children’s receptive vocabulary was 99.79 (*SD = *11.71) out of a possible score of 140.

#### 2.4.4 Child's working memory

Also in Visit 2, the *Visuo‐Spatial Sequencing (VSS)* task from the Amsterdam Neuropsychological Tasks (ANT) was used to measure visuo‐spatial *working memory* (de Sonneville, 1999). In this task, children were presented with a grey square containing nine circles symmetrically positioned in a 3x3 matrix on a computer screen. After a beep, a sequence of circles was pointed at by a computer animated hand, and after the sequence the children took control of the mouse to replicate the sequence of circles. The test consisted of 24 trials, and gradually increased in difficulty in the number of targets and complexity of the sequence. Working memory was assessed using the total number of correct targets in the correct order, with a total of 100 possible correct targets. Data were available for 201 (97%) of children with interaction data. The mean score for correct targets in the correct order was 66.47 (*SD = *17.61).

#### 2.4.5 Child's second‐order false belief

**Figure 1:**
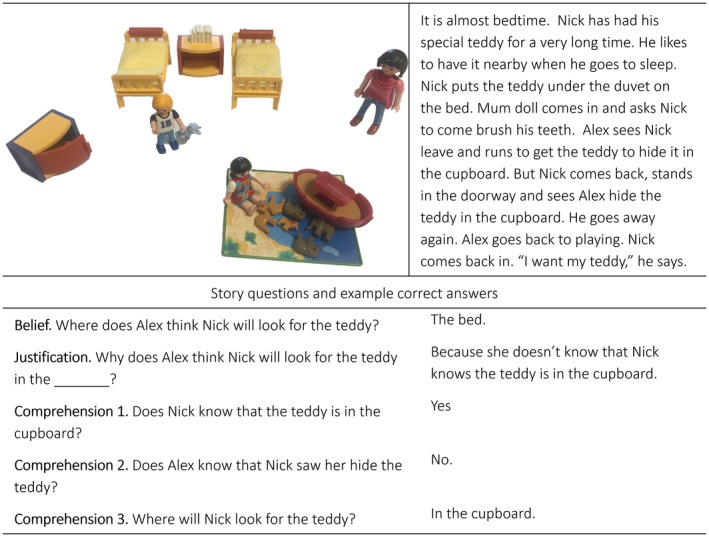
Second‐order false belief story. This is an example of the story for male participants, whereby the protagonist was gender matched and named “Nick”. The protagonist for female participants was named “Kate”

In the first visit, children were told a *second‐order false belief* story (Paine et al., 2018), adapted from other second‐order belief paradigms (Perner & Wimmer, 1985). The story was enacted with plastic Playmobil figures by the experimenter (see Figure 1). Children had to answer all belief, justification, and comprehension questions correctly to be classified as passing second‐order false belief. Data were available for 200 (97%) of children with interaction data.

#### 2.4.6 Mother–child interaction in early infancy

**Figure 2:**
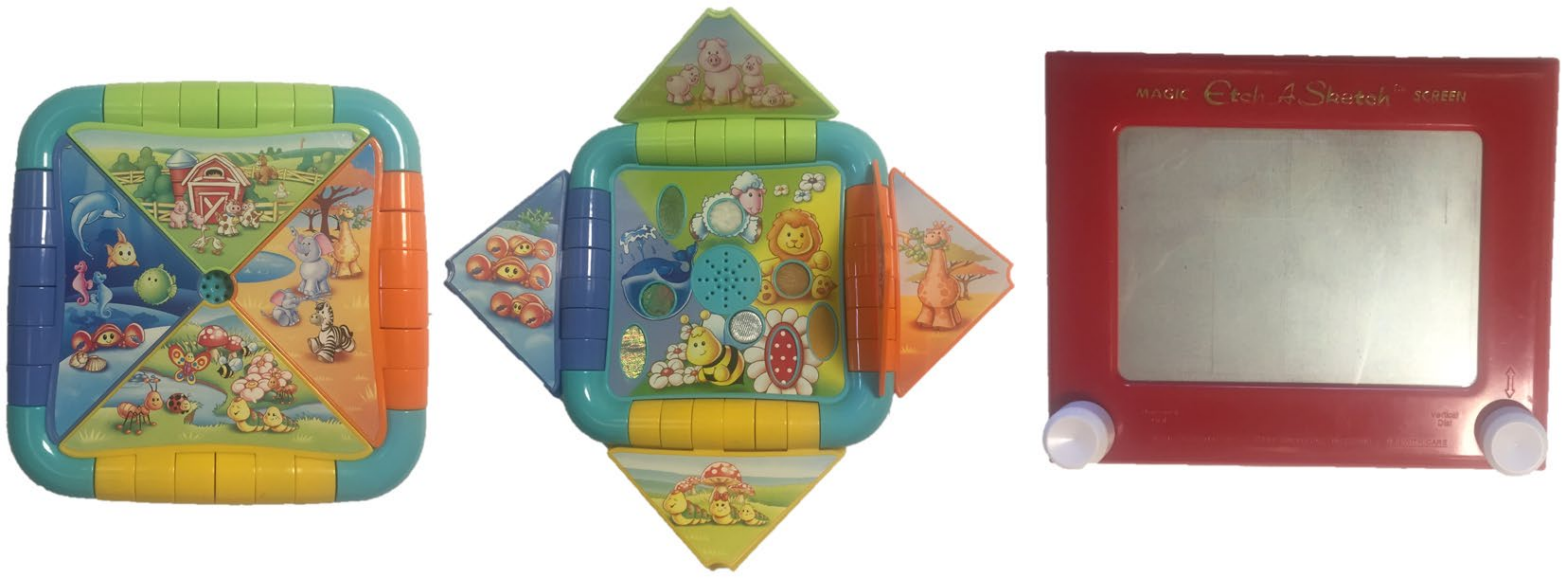
Activity board used in the early infancy mother–child topic sharing task (left) and Etch‐a‐Sketch used in middle childhood mother–child topic sharing task (right)

Mothers and their firstborns were given a topic sharing task using an activity board, a commercially available plastic toy with flaps that equates to a wordless picture book. Pilot testing showed that a toy rather than a book was more acceptable to parents. Similar wordless picture books have been used in previous research to elicit discourse between parents and their children (e.g., Ruffman et al., 2002). The activity board contained pictures of cartoon animals from farm, safari, park, and under the sea themes on flaps that could be opened and closed (Figure 2). The activity board was presented to the mother and infant as they were seated on the sofa or floor, and each mother was then asked to show the infant the toy. This mother–infant interaction was video‐recorded for 2 min and took place in the mother’s choice of language.

#### 2.4.7 Mother–child interaction in middle childhood

In Visit 2 of the middle childhood assessment, the mother‐firstborn dyads were observed as they played with an Etch‐a‐Sketch, a commercially available drawing toy (Figure 2). The mother and child were assigned one dial of the toy each to use; one which creates vertical lines and the other that creates horizontal lines. Using their dials at the same time, it is possible to produce diagonal lines. After 1 min of free play with the Etch‐a‐Sketch, the dyad were given 3 min to attempt to draw a house (Stevenson‐Hinde & Shouldice, 1995), which was video‐recorded. Interactions took place in the dyad’s choice of language.

#### 2.4.8 Child's free play in middle childhood

**Figure 3:**
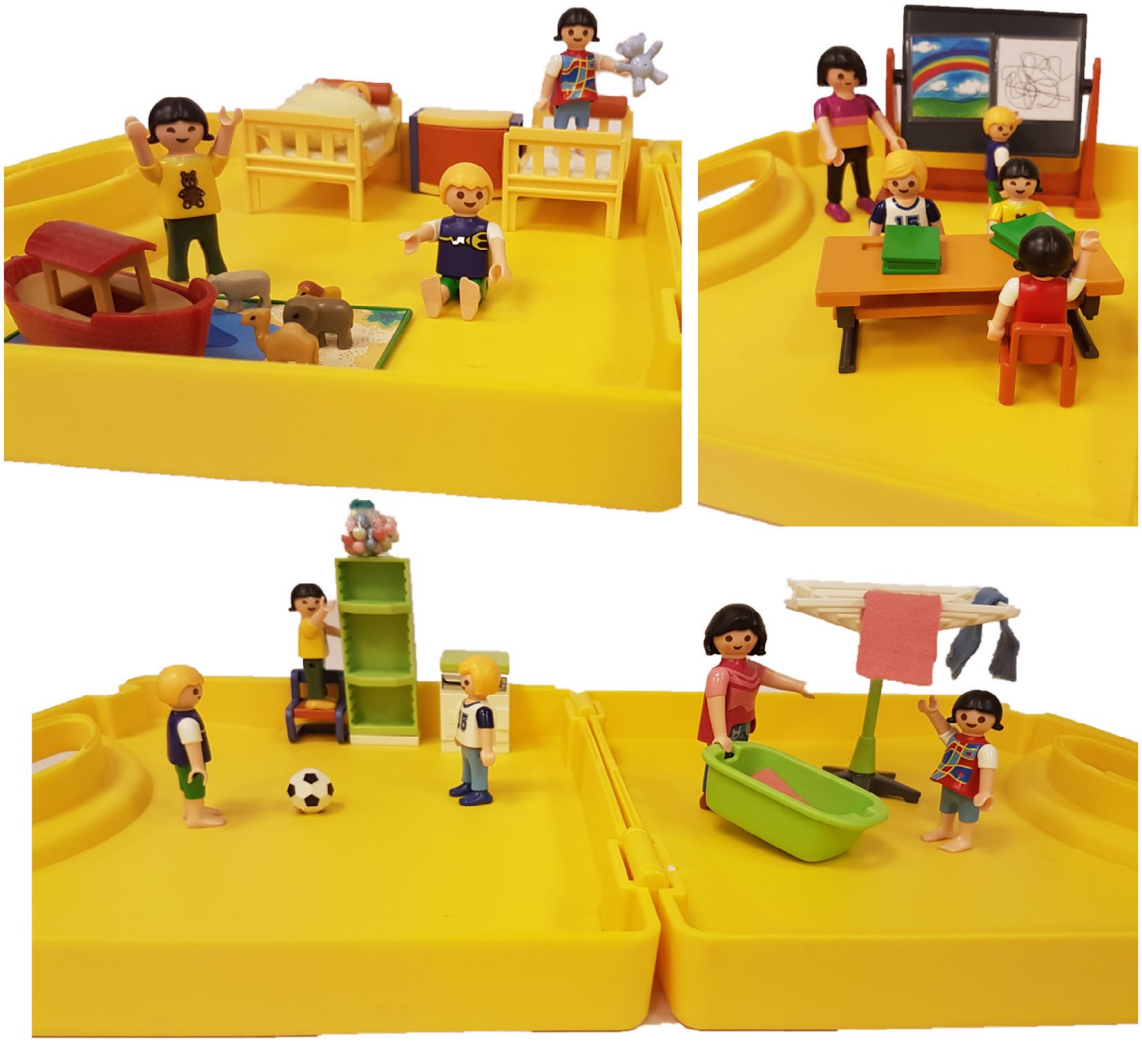
Playmobil set presented in the middle childhood free play task

In Visit 1, children were individually administered a standardized battery of tasks enacted using Playmobil figures designed to assess aspects of their social understanding (Paine et al., 2018). Following the last task, the children were video‐recorded as they were given the opportunity to play with the Playmobil figures in any way that they would like, for at least 3 min (see Figure 3). Experimenters were encouraged not to prompt the children’s play and only participate in the play at the child’s request.

#### 2.4.9 Transcripts of speech

The video recordings of the early infancy and middle childhood interactions, in addition to the middle childhood free play task, were transcribed in 5‐s time segments (24 segments for the early infancy interaction, and 36 segments for the middle childhood interaction and child free play task). Although the majority of parent–child interactions were carried out in English, trained translators transcribed tasks that took place in Welsh, Spanish, French, Dutch, and German. Coded transcripts that were under the full task length were prorated up to the full number of segments, by dividing codes by available segments and multiplying them by expected segments. At the early infancy assessment, three cases could not be transcribed due to video technical errors. At the middle childhood assessment, one case could not be transcribed due to background noise. For the free play task in middle childhood, data were unavailable for five cases (three children refused; one case could not be translated; and in one case there was a technical error).

#### 2.4.10 Coding of speech

Relationships between mothers’ references to internal states and children’s understanding of minds could be explained by mothers’ *talkativeness* as a measure of their engagement in the task rather than the specific content of mothers’ speech. Therefore, mothers’ talkativeness during the interaction task was computed by dividing the number of 5‐s segments containing speech by total number of 5‐s segments in the task, generating proportional talkativeness scores between 0 and 1. This measurement of talkativeness has been validated by *Audacity* voice analysis software measuring the overall production of maternal speech in 32% of the mother–infant dyads (Roberts et al., 2013). The mean proportional score for mothers’ talkativeness was .87 (*SD = *0.18) in early infancy and .82 (*SD *= 0.14) in middle childhood.

All 5‐s segments in the transcripts of speech were coded for the use of *ISL* by: (a) mothers during the early infancy interaction task (2 min); (b) mothers during the middle childhood interaction task (3 min); (c) children during the middle childhood interaction task (3 min); and (d) children during the middle childhood free play task (3 min). ISL was coded using an expanded version of the coding scheme used by Roberts and colleagues (2013). ISL was divided into seven categories: *Perception*, *physiology*, *preference*, *intention*, *desire*, *emotion*, and *cognition* (Table 1). Mothers’ references to internal states in the mother–child interaction in middle childhood were also coded for the referent (self or child). Children’s references to internal states during the free play task in middle childhood were also coded for the referent, that is, being about themselves or about others (toy character or any other present/non‐present individual).

**Table 1:** Internal state language coding scheme

Internal state category	Description
Perception	Comments made about perception of an object using one of five senses, such as “see,” “hear,” “feel,” “taste,” “smell.”
Physiology	Comments made about physical states and sensations, including “sleepy,” “pain,” “hot/cold (as in temperature),” “sick,” “comfy.”
Preference	Comments made about positive or negative judgements of an object, action or experience. Coding preference includes terms include “like,” “hate,” “love,” “favourite,” “enjoy,” “interest.”
Intention	Comments made about present intentional actions that are goal‐directed and future intentions. Includes “try,” “attempt,” “on purpose,” “mean to,” “going to.”
Desire	Comments made about longing for an object, action or experience. Desire terms include “want,” “wish,” “hope,” “fancy,” “rather,” “need (as in want).”
Emotion	Comments made about feeling states, including basic emotions “happy,” “sad,” “surprised,” “disgusted” and variations like “fed up,” “bored,” “glad,” “excited.”
Cognition	Comments made about beliefs and knowledge. Also include general terms indicating other cognitive activity, such as “remember,” “imagine,” “pretend,” “understand.”

An independent observer coded mothers’ and children’s ISL for 65 (31.4%) mother–child interactions in early infancy and middle childhood. Excellent inter‐rater reliability was established (median *ICC *= .99 for mothers’ speech and .94 for children’s speech). Similarly, inter‐rater relability was established for ISL during the free play task for another subsample of 67 (32.4%) children (median *ICC *= .95). The summary measures of ISL in each task were: (a) counts of all references to internal states; (b) counts of categories of ISL; and (c) counts of ISL categories by referent (all based on rate per minute).

## 3 RESULTS

### 3.1 Data analysis

We first describe features of children’s social understanding in middle childhood and mothers’ use of ISL at two time points of assessment. Nonparametric analyses were used to investigate associations for speech variables as the data were not normally distributed. Associations that reached *p* < .05 significance were followed up with regression analyses. The child’s references to their own and others’ cognitions during the free play were dichotomized for subsequent logistic regression analyses due to a high frequency of zero scores, which violated the assumption of normality for multiple regression.

An iterative approach was taken to test associations between maternal ISL and children’s social understanding while controlling for relevant covariates of mothers’ ISL and children’s performance on the social understanding measures. We examined (a) univariate prediction of children’s understanding from mothers’ ISL alone; then (b) controlled for family and maternal covariates; and finally (c) retained relevant family and mother covariates and controlled for child‐related covariates. Analyses of all predictor variables in the models showed no collinearity (variance inflation factor < 10, tolerance > .20; Myers, 1990).

### 3.2 Children's social understanding skills in middle childhood

#### 3.2.1 Second‐order false belief

Fifty‐seven (27.5%) children passed the second‐order false belief task. More girls than boys passed, but the difference only approached statistical significance *χ* ^2^(1) = 3.64, *p *= .06. Passing second‐order false belief was not associated with the child’s age or working memory, but was associated with higher verbal ability scores and lower exposure to sociodemographic adversity (*p*s < .01, Table 2). Children who passed second‐order false belief were more likely to refer to cognitions *t*(197) = 2.03, *p *= .04 during the mother–child interaction (mean cognitions for those who passed = 0.21, *SD *= 0.30 vs. 0.12, *SD *= 0.26 for those who did not).

**Table 2:** Descriptive statistics and intercorrelations among key variables of interest

	1	2	3	4	5	6	7	8	9	10	11	12	13	14	15	16	17	18	19	20
1. sociodemographic adversity	–																			
2. Mothers’ verbal ability (WTAR)	−.56**	–																		
3. Mother early infancy talkativeness†	−.10	−.08	–																	
4. Mother total ISL early infancy†	−.26**	.07	.36**	–																
5. Mother middle childhood talkativeness†	−.12	.00	.10	.20**	–															
6. Mother total ISL middle childhood†	−.23**	.15*	.01	.34**	.44**	–														
7. Mother total cognitions middle childhood†	−.24**	.18*	.08	.32**	.35**	.78**	–													
8. Mother self cognitions middle childhood†	−.14*	.16*	.04	.21**	.23**	.59**	.82**	–												
9. Mother child cognitions middle childhood†	−.25**	.15*	.04	.29**	.32**	.68**	.78**	.35**	–											
10. Child age (months)	.11	−.06	.01	.17*	−.23**	−.32**	−.21**	−.15*	−.19**	–										
11. Child gender	−.19**	.07	.06	−.06	.07	.02	.02	.02	−.02	.01	–									
12. Child receptive vocabulary (BPVS)	−.43**	.41**	.13	.25**	.08	.18**	.22**	.16*	.19**	−.19**	.09	–								
13. Child working memory (VSS)	−.20**	.16*	.17*	.10	.02	−.01	.03	.02	.01	.26**	.22**	.26**	–							
14. Child total ISL middle childhood†	.06	.04	.00	−.07	−.05	.11	.11	.17*	.03	.06	.05	.03	−.06	–						
15. Child total cognitions middle childhood†	−.02	.02	.05	−.03	−.10	.10	.17*	.24**	.06	.01	−.04	.04	.01	.55**	–					
16. Child total ISL middle childhood freeplay	−.06	.01	.01	−.01	.06	.02	.09	.17*	−.03	.02	.06	.05	−.05	.03	.05	–				
17. Child total cognitions middle childhood freeplay	−.01	−.04	.05	−.06	.09	.01	.08	.10	.01	.01	.06	−.01	−.07	.02	.06	.65	–			
18. Child cognitions self middle childhood freeplay	.05	−.05	.02	−.11	.12	−.05	−.01	.00	−.03	.06	.06	−.06	−.04	−.08	.01	.48**	.78**	–		
19. Child cognitions other middle childhood freeplay	−.04	.00	.09	.03	−.02	.13	.18*	.23**	.08	−.06	.04	.08	−.07	.08	.10	.41**	.60**	.05	–	
20. Child second‐order false belief	−.19**	.07	.00	.09	.01	.12	.19*	.16*	.09	.08	.13	.21**	.03	.07	.17*	.01	−.01	.00	−.02	–
*Mean*	−0.15	97.92	.87	1.77	.82	1.77	0.71	0.36	0.35	82.88	.45	99.79	66.47	0.43	0.14	1.13	0.37	0.23	0.13	.29
*Standard deviation*	0.92	14.20	0.18	1.51	0.14	1.43	0.89	0.50	0.55	4.44	0.50	11.71	17.61	0.45	0.27	0.93	0.46	0.36	0.25	0.45

Note

ISL; Internal State Language.

^†^ Denotes features of language within mother–child interaction. Means and standard deviations for references to internal states are based on counts per minute. Associations including non‐normal variables were tested with Spearman’s rho, and associations between dichotomous variables were tested by Kappa coefficients.

* *p *< .05,

** *p *< .01.

#### 3.2.2 Children's references to internal states during free play

Descriptive data for children’s total use of ISL during free play with the Playmobil figures is presented in Table 2. Most children made at least one reference to an internal state during free play (*n = *166, 83%), referring most often to cognitions during their free play (Table 3). Children referred to their own cognitions significantly more than the cognitions of others *t*(201) = 3.22, *p *< .001. Features of children’s ISL during free play were not significantly associated with passing the second‐order false belief task (all *ps *> .05, see Table 2).

**Table 3:** Descriptive statistics showing categories of mother and child references to internal states

	Mother				Child			
	Early infancy interaction		Middle childhood interaction		Middle childhood interaction		Middle childhood free play	
	*M*	*SD*	*M*	*SD*	*M*	*SD*	*M*	*SD*
Perception	0.67	0.96	0.09	0.24	0.04	0.15	0.15	0.28
Physiology	0.03	0.13	0.01	0.05	0.00	0.02	0.05	0.16
Preference	0.26	0.41	0.02	0.08	0.01	0.08	0.06	0.20
Intention	0.17	0.39	0.49	0.60	0.12	0.24	0.25	0.39
Desire	0.43	0.59	0.43	0.51	0.10	0.22	0.23	0.38
Emotion	0.01	0.09	0.02	0.10	0.00	0.02	0.03	0.11
Cognition	0.20	0.42	0.71	0.89	0.14	0.27	0.37	0.46

Note

Means (*M*) and standard deviations (*SD*) for references to internal states are based on counts per minute.

### 3.3 Mothers’ references to internal states

All mothers spoke to the child during the interaction task at the middle childhood assessment and the majority did so in early infancy. The majority of mothers produced at least one reference to internal states at the early infancy assessment (*n = *168, 83%) and at the middle childhood assessment (*n = *182, 88%). Mothers’ speech about internal states showed significant continuity over time. The mother’s references to internal states in early infancy were positively associated with her use of ISL during middle childhood (*p *< .001). Mothers referred more often to the child’s internal states than their own in early infancy and in middle childhood (early infancy *M* = 1.63 *SD* = 1.44, middle childhood *M* = 1.24, *SD* = 1.12). However, mothers made more references to their own internal states at the middle childhood interaction (early infancy *M* = 0.14, *SD* = 0.34, middle childhood *M* = 0.52, *SD* = 0.62). There were also changes over time by category. At early infancy, mothers referred most often to perception, desires, and preferences, whereas by middle childhood they referred most often to cognitions, intentions, and desires. Mothers’ references to cognitions and intentions significantly increased (*p*s < .001) and references to perception, physiology, and preferences significantly decreased (*ps *< .05; Table 3).

#### 3.3.1 Association with family and maternal characteristics

Mothers who referred to more internal states in early infancy were more talkative and had lower exposure to sociodemographic risk. Mothers’ ISL in early infancy was not associated with her verbal ability (Table 2). At the middle childhood assessment, mothers who experienced less family sociodemographic adversity referred to internal states more often. Mothers’ ISL in middle childhood was positively associated with measures of verbal behaviour, including general talkativeness during the task and verbal ability (all *p*s < .05).

#### 3.3.2 Associations with child characteristics

Mothers’ total references to internal states did not differ as a function of the child’s gender at either time point; at the middle childhood assessment, mothers were more likely to make references to internal states to younger children and those with higher verbal ability scores (all *ps *< .01). Mothers’ references to cognitions during middle childhood were associated with children’s own tendencies to talk about cognition within the interaction (*p *= .02, Table 2).

### 3.4 Relationships between mothers’ references to internal states and children's social understanding

Mothers’ overall propensities to refer to internal states and individual categories of ISL during interactions in early infancy and middle childhood were examined in relation to the two measures of children’s social understanding (see Table 2). Mothers’ overall references to internal states in early infancy were unrelated to features of children’s social understanding in middle childhood (see Table 2), as were mothers’ individual categories of ISL (all *p*s > .05).

However, in middle childhood, mothers’ specific references to cognitions were associated with children’s passing of the second‐order false belief task and children’s references to cognitions during the free play task (*ps *< .05). No other categories of mothers’ ISL were associated with children’s social understanding (all *p*s > .05). When mothers’ and children’s references to cognitions were separated according to referent of the cognitive terms, mothers’ references to her *own* cognitions remained significantly and positively associated with children’s second‐order false belief (*p* = .02) and references to *others’* cognitions during free play (*p* < .001).

#### 3.4.1 Mothers’ references to her own cognitions during the middle childhood interaction and children's second‐order false belief

Mothers’ references to her own cognitions were associated with a twofold increase in children’s understanding of second‐order false belief (Table 4, Model a). When family and maternal correlates of second‐order false belief and mothers’ ISL during early infancy were controlled, mothers’ references to her own cognitions remained significantly associated with children’s passing of second‐order false belief. Lower exposure to family sociodemographic adversity was also associated with a greater likelihood of children passing the second‐order false belief task *χ* ^2^(5) = 17.34, *p *= .008, Nagelkerke *R* ^2 ^= .14 (Model b). When sociodemographic adversity and child factors were entered into the final model (Model c), mothers’ references to her own cognitions represented a significant step in the model *χ* ^2^(1) = 6.19, *p *= .01, final model *χ* ^2^(5) = 27.79, *p *< .001, Nagelkerke *R* ^2 ^= .19. Sociodemographic adversity, child age, and child verbal ability were significantly related to children’s understanding of second‐order false belief. Mothers’ references to her own cognitions during the mother–child interaction were associated with a significant twofold increase in children’s passing of the second‐order false belief task, Wald statistic = 6.16, *p *= .01, *OR *= 2.24, 95% *CI*(1.19–4.24).

**Table 4:** Prediction of children's passing of second‐order false belief from mothers’ frequency of references to her own cognitions during middle childhood interaction, controlling for family, mother, and child characteristics

Model (a)				Model (b)				Model (c)			
Predictor	*R* ^2^	*e β*	95% CI for OR	Predictor	*R* ^2^	*e β*	95% CI for OR	Predictor	*R* ^2^	*e β*	95% CI for OR
	.05**				.14**				.19***		
Constant		0.30				3.63				0.00	
*Step 1*				*Step 1*				*Step 1*			
Mother references to own cognitions during middle childhood interaction		2.20**	1.22–3.98	Sociodemographic adversity		0.45**	0.25–0.81	Sociodemographic adversity		0.64*	0.41–1.01
				Mother verbal ability		0.98	0.95–1.01				
				Mother talkativeness in middle childhood		0.23	0.02–3.75				
				Mother ISL during early infancy interaction		1.03	0.82–1.30				
				*Step 2*				*Step 2*			
				Mother references to own cognitions during middle childhood interaction		2.65**	1.33–5.28	Child age		1.12**	1.03–1.21
								Child receptive vocabulary		1.04**	1.01–1.08
								Child references to cognitive states in mother–child interaction		3.05	0.74–9.89
								*Step 3*			
								Mother references to own cognitions during middle childhood interaction		2.24**	1.19–4.24

Note

Logistic regressions. ISL; Internal State Language. The coefficients presented are those obtained in the final model. *R* ^2^ represents Nagelkerke statistic.

* *p *< .05,

** *p *< .01,

*** *p *< .001.

#### 3.4.2 Mothers’ references to her own cognitions during the middle childhood interaction and children's references to others’ cognitions in free play

Mothers’ references to her own cognitions were related to a twofold increase in children’s likelihood of talking about the cognitions of others during their free play (Table 5, Model a). This finding was investigated whilst controlling for family and maternal factors (Model b) and finally child factors (Model c) associated with mothers’ use of ISL. Mothers’ references to her own cognitions represented a significant step in the final model *χ* ^2^(1) = 6.75, *p *= .018, final model *χ* ^2^(4) = 11.87, *p *= .009, Nagelkerke *R* ^2 ^= .08. Mothers’ references to her own cognitions during the mother–child interaction were associated with a significant twofold increase in children’s references to others’ cognitions during free play, Wald statistic = 6.67, *p *= .01, *OR *= 2.22, 95% *CI*(1.21–4.09).

**Table 5:** Prediction of children's references to others’ cognitions during freeplay from mothers’ frequency of references to her own cognitions during middle childhood interaction, controlling for family, mother, and child characteristics

Model (a)				Model (b)				Model (c)			
Predictor	*R* ^2^	*e β*	95% CI for OR	Predictor	*R* ^2^	*e β*	95% CI for OR	Predictor	*R* ^2^	*e β*	95% CI for OR
	.06**				.06**				.08*		
Constant		0.26				6.34				0.21	
*Step 1*				*Step 1*				*Step 1*			
Mother references to own cognitions during middle childhood interaction		2.43**	1.33–4.43	Sociodemographic adversity		0.77	0.47–1.27	Child age		0.99	0.92–1.07
				Mother verbal ability		0.98	0.95–1.01	Child receptive vocabulary		1.01	0.98–1.04
				Mother talkativeness in middle childhood		0.19	0.02–2.35	Child references to cognitive states in mother–child interaction		2.56	0.84–7.77
				Mother ISL during early infancy interaction		0.99	0.78–1.25				
				*Step 2*				*Step 2*			
				Mother references to own cognitions during middle childhood interaction		2.23*	1.16–4.28	Mother references to own cognitions during middle childhood interaction		2.22**	1.21–4.09

Note

Logistic regressions. ISL; Internal State Language. The coefficients presented are those obtained in the final model. *R* ^2^ represents Nagelkerke statistic.

* *p *< .05,

** *p *< .01.

## 4 DISCUSSION

Mothers’ references to internal states were associated with children’s social understanding in middle childhood. Children who heard more frequent references to their mothers’ cognitions (e.g., “I don't even *know* how to do a circle!”) were more likely to pass a second‐order false belief task and talk spontaneously about other people’s cognitions (e.g., “Kate doesn't *know* where it is.”). This association remained significant when covariates such as sociodemographic adversity, the child’s age and verbal ability, and the mother’s verbal ability and earlier speech about internal states were taken into account. This pattern of results corroborates previous findings showing positive associations between mothers’ references to cognitive terms in picture book reading tasks and performance on strange stories tasks at ages 7 and 10 (Adrian et al., 2007; Ensor et al., 2014). We have now extended these findings by demonstrating the importance of mothers’ use of cognitive terms for other measures of children’s social understanding in middle childhood.

The detailed exploration of the referents of mothers’ speech about cognition extends findings of previous work investigating mothers’ ISL in relation to children’s advanced understanding of minds (e.g., Ensor et al., 2014). Mothers’ references to her own, not the child’s, cognitions drove the association between her talk about internal states and children’s understanding of second‐order false belief. Furthermore, mothers’ references to her own cognitions were associated with a twofold increase in children’s tendency to talk spontaneously about the thoughts and knowledge of others, for example, attributing internal states to the characters in their play narratives (e.g., “Her mum doesn't *believe* her ‘cause Alex is still in bed having a snooze.”). These findings suggest that a mother’s discussions about her own thoughts may contribute to older children’s social understanding by offering the child insight into another person’s perspective (Harris, 1999). Mothers’ references to her own cognitions may provide children with a “window” into another person’s unobservable, mental processes, and are therefore associated with children’s developing capacities to understand and communicate thoughts about thoughts.

The present findings also extend knowledge about the association between different measures of children’s social understanding in middle childhood. Children’s understanding of second‐order false belief was associated with their own references to cognitions when interacting with their mothers, but not during free play. The mixed findings from other studies investigating links between children’s social understanding tasks and mental state references in middle childhood (Longobardi et al., 2014) therefore may be partly due to differences in the contexts where children’s ISL is studied. This finding may also be driven by thought‐laden conversations elicited by the mother. Further investigation of children’s ISL across a variety of contexts is warranted.

Despite the growing body of work exploring the influence of mothers’ ISL on children’s developmental outcomes, it is surprising that there are so few studies that have examined the patterns of change and continuity in mothers’ references to internal states (Ensor et al., 2014). Our inclusion of mothers’ references to internal states in early infancy revealed that mothers were consistent in their use of ISL over time, despite age‐appropriate changes in its content. This finding extends studies showing stability over 1 and 2 years (Ruffman et al., 2002). Although some research has found mothers’ use of ISL to be less consistent over longer periods of time (Ensor et al., 2014), such research does not include coding of inner states most prominent in the early years, such as perception (Slaughter, Peterson, & Carpenter, 2008). Indeed, we identified qualitative shifts in mothers’ ISL over time, corroborating early findings that mothers’ categories of ISL change over time, as does the referent of mothers’ ISL (Beeghly et al., 1986). We have now extended these findings across a larger time interval.

Despite continuity over time in mothers’ propensity to refer to internal states, it is worthy of note that mothers’ ISL at 6 months did not predict features of children’s social understanding at 7 years. Thus it is not simply mothers’ general tendencies to speak about the mind but rather their specific input at the age of seven that promoted social understanding at that age. It is possible that children’s development of advanced understanding of minds is fostered by speech about abstract internal states that increasingly enters the mother’s vocabulary as the child develops (Beeghly et al., 1986). More longitudinal work including earlier measures of children’s social understanding is needed.

Our study has limitations. This study explored ISL within very concentrated time periods; recording speech in natural observations of families over longer periods of time would probably yield a more comprehensive measure of children’s daily exposure to ISL. However, the time‐consuming nature of that method inevitably would result in a trade‐off with sample size. Our procedure for home observation was devised to provide some balance between the issues of time, sample size, and richness of data, especially in terms of the inclusion of numerous covariates such as mothers’ language ability and various child characteristics.

Furthermore, it is worthy of note that the sociodemographic adversity index used in the present study was constructed from maternal variables; certainly, a more encompassing measure of adversity might also include information about fathers. However, the maternal variables included in the adversity index are key risk factors for children’s outcomes that could be measured, whether or not the father chose to participate in the study.

The use of a single second‐order false belief task is also a limitation, given that this task alone does not give a full picture of what children are capable of in middle childhood. However, test–retest reliability of similar second‐order tasks is reported as acceptable and therefore single theory‐of‐mind tasks are considered acceptable for research purposes (Hughes et al., 2000). Furthermore, the association between children’s spontaneous ISL during the mother–child interaction and their performance on the structured task indicates some convergent validity between directly observed and experimental measures of children’s social understanding. In future work, it would be helpful to use additional false belief tasks in a battery of age‐appropriate measures of social understanding.

The evidence presented here provides a platform on which future studies can investigate further possibilities whereby children’s social understanding abilities could be promoted in middle childhood. Recent studies demonstrated that training programmes encouraging mind‐related discourse within children’s peer groups result in improved performance on social understanding tasks (e.g., Lecce, Bianco, & Banerjee, 2016). Children’s social understanding abilities might also be fostered via promoting ISL within mother–child interactions (Aram, Fine, & Ziv, 2013). Our findings suggest that encouraging mothers to discuss *her own* internal states with her child may be effective in promoting older children’s advanced understanding of minds. Furthermore, given that sociodemographic adversity was associated with mothers’ ISL and children’s social understanding, our findings suggest that such interventions might be of most benefit to families that come from sociodemographic disadvantage.

In summary, the present study highlighted the ongoing importance of children’s conversations about internal states with their mothers in middle childhood. We have described the nature of internal state references within the mother–child dyad in this overlooked age range and demonstrated that continued exposure to conversations about people’s thoughts in middle childhood is important for children’s developing understanding of minds.

## CONFLICT OF INTEREST

The authors of this manuscript have no conflicts of interest to declare.
